# Synthetic dimensions in ultracold polar molecules

**DOI:** 10.1038/s41598-018-21699-x

**Published:** 2018-02-21

**Authors:** Bhuvanesh Sundar, Bryce Gadway, Kaden R. A. Hazzard

**Affiliations:** 1 Department of Physics and Astronomy, Rice University, Houston, TX 77251 USA; 2 Rice Center for Quantum Materials, Rice University, Houston, TX 77251 USA; 3 Department of Physics, University of Illinois at Urbana Champaign, Urbana, IL 61801 USA

## Abstract

Synthetic dimensions alter one of the most fundamental properties in nature, the dimension of space. They allow, for example, a real three-dimensional system to act as effectively four-dimensional. Driven by such possibilities, synthetic dimensions have been engineered in ongoing experiments with ultracold matter. We show that rotational states of ultracold molecules can be used as synthetic dimensions extending to many – potentially hundreds of – synthetic lattice sites. Microwaves coupling rotational states drive fully controllable synthetic inter-site tunnelings, enabling, for example, topological band structures. Interactions leads to even richer behavior: when molecules are frozen in a real space lattice with uniform synthetic tunnelings, dipole interactions cause the molecules to aggregate to a narrow strip in the synthetic direction beyond a critical interaction strength, resulting in a quantum string or a membrane, with an emergent condensate that lives on this string or membrane. All these phases can be detected using local measurements of rotational state populations.

## Introduction

Ultracold polar molecules offer unique possibilities for creating strongly correlated matter, owing to their strong anisotropic long-ranged dipolar interactions and their complex rotational and vibrational structure^1–14^. Although previous experimental and theoretical research has utilized the rotational degree of freedom^9–21^, it has used only a few rotational or dressed rotational states.

In this article, we propose to use rotational states of polar molecules as a synthetic dimension, which can have up to hundreds of synthetic lattice sites. The synthetic tunnelings are driven by microwaves resonant with rotational state transitions. This gives rise to a system with a fully tunable synthetic single particle Hamiltonian, which experiments can use to realize arbitrary synthetic band structures, including topological ones. We show that dipole interactions in polar molecules lead to interesting phases, even without any special engineering or fine tuning. For example, we show that molecules frozen in a periodic real space array undergo a spontaneous dimensional reduction, forming a fluctuating quantum string or membrane. At strong interactions, the string/membrane hosts an emergent condensate of hardcore bosons. We show that ongoing experiments can realize and probe these strings/membranes and condensate.

**Figure 1:**
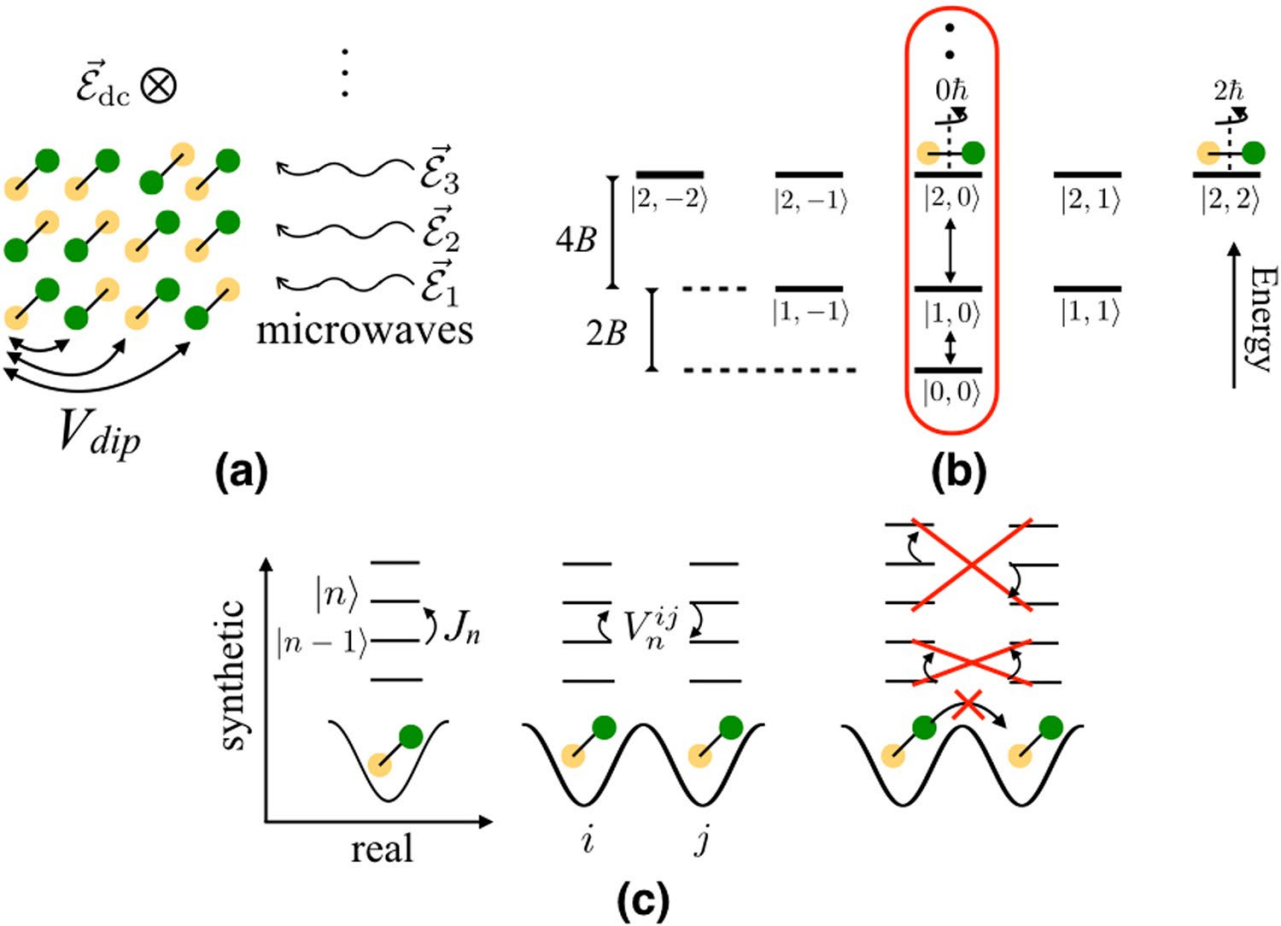
(**a**) Schematic of setup. Molecules in a periodic (1D or 2D) array in the *x*-*y* plane are driven by microwaves, and interact via long-ranged dipole interactions. (**b**) A synthetic dimension is formed by the rotational states circled in red, in addition to the real spatial dimensions. Vertical arrows indicate transitions driven by microwaves (see also (**c**)). Here, $|n,m\rangle$ refers to a rotational state with total and azimuthal angular momentum *n* and *m*. (**c**) Two types of processes occur in this system: Microwaves ${\overrightarrow{ {\mathcal E} }}_{n}$ drive effective tunneling ${J}_{n}\propto |{\overrightarrow{ {\mathcal E} }}_{n}|$ (left), and correlated tunneling ${V}_{n}^{ij}$ arise from dipole interactions between molecules at real lattice sites *i* and *j* (middle). Off-resonant processes (right) disappear in the rotating-wave approximation. Dipole-induced transitions to states outside the circled set in (**b**) are also made off-resonant by a static electric field.

Researchers have created nearly quantum degenerate gases of several heteronuclear molecular species, such as KRb, NaRb, NaK, and RbCs, in their ground state^22–26^. All of these have a strong electric dipole moment of about a Debye. These molecules also have a large number of rotational quantum states. We define a synthetic lattice, whose sites are a subset of a molecule’s rotational states. To create a large synthetic lattice, we propose to shine several microwaves in parallel to drive transitions up to a highly excited rotational state, as illustrated in Fig. 1. These transitions correspond to tunneling in the synthetic lattice. Experimentalists can simultaneously apply a large number of microwaves with fully controllable amplitudes, phases, and frequencies ranging from a few to several tens of GHz using commercially available technology (see Supplementary Materials).

Experimentalists have created synthetic dimensions in other ultracold gases from their motional^27,28^, spin^29–32^, clock^33–35^, or rotational^36^ states that are coupled by Raman lasers, analogous to our proposal’s coupling of rotational states with microwaves. Our proposal shares some features with these other methods. We can fully control every tunneling amplitude and on-site potential by tuning the microwaves’ complex amplitudes and detunings. By appropriate choice of the rotational states, we can impose periodic or open boundaries on the synthetic lattice, or create other spatial topologies. We can image populations in the synthetic lattice with single-site resolution.

Additionally, realizing synthetic dimensions in polar molecules has significant advantages over other systems. First, the experimentally feasible size of the synthetic dimension is orders of magnitude larger. Second, since the internal states are directly coupled via microwaves without an intermediate excited state, the system does not suffer from heating encountered in schemes that employ two-photon Raman processes. Third, our system is insensitive to magnetic field noise that limits other methods. Finally, strong dipole interactions lead to rich many-body physics at a favorable energy scale.

## Setup

We consider a unit-filled periodic array of molecules trapped in the *x*-*y* plane in an optical lattice or a microtrap array^37–40^, as illustrated in Fig. 1. Current experiments achieve ~25% filling^41^, and experimental advances are steadily increasing this number. We impose a sufficiently deep lattice to completely suppress tunneling in real space. This avoids problematic molecular reactions^18,42–46^ or complicated collision processes^47–52^ that occur if two molecules occupy a single lattice site.

To create a 1D synthetic lattice with *N*_rot_ sites and open boundaries, the molecules are driven by *N*_rot_ − 1 microwaves, as shown in Fig. 1. The polarization of the microwaves is chosen to yield the desired sign for the amplitude of angular momentum exchange driven by the dipole interaction. As we will show, microwaves that are linearly polarized in the *z* direction yield a positive sign, while microwaves with a left-circular polarization with respect to the *z* axis yield a negative sign. For linearly polarized microwaves, the synthetic lattice sites lie in the $|n,0\rangle$ subspace of the single-molecule rotational eigenstates, with $\hat{z}$ the quantization axis. In this case, the *n*^th^ microwave is resonant with the transition from $|n-1,0\rangle$ to $|n,0\rangle$. We apply a small electric field to detune the $|n,m\ne 0\rangle$ states, so that molecules remain in the $|n\mathrm{,0}\rangle$ space. The detuning due to the electric field is larger than dipole interactions and hyperfine mixing even for moderate electric fields ~${\mathscr{O}}\mathrm{(10)}$ V/cm^53^. For left-circularly polarized microwaves, the synthetic lattice sites lie in the $|n,n\rangle$ subspace, and a small electric field detunes away the $|n,m\ne n\rangle$ subspace. We discuss the technical details for applying the microwaves and accuracy of the resulting effective Hamiltonian in more detail in the Supplementary Material.

In the rotating wave approximation, our system is described by

1 $$\hat{H}=-\sum _{nj}\,{J}_{n}{\hat{c}}_{n-\mathrm{1,}j}^{\dagger }{\hat{c}}_{nj}+\sum _{nij}\,{V}_{n}^{ij}{\hat{c}}_{n-1,i}^{\dagger }{\hat{c}}_{ni}{\hat{c}}_{nj}^{\dagger }{\hat{c}}_{n-1,j}+{\rm{h.c.}},$$

where ${\hat{c}}_{nj}$ (${\hat{c}}_{nj}^{\dagger }$) annihilates (creates) a molecule on the real lattice site *j* and synthetic lattice site *n*. Here, *J*_*n*_ is the synthetic tunneling amplitude induced by a resonant microwave, and ${V}_{n}^{ij}$ is the angular momentum exchange amplitude induced by dipole interaction. When the synthetic lattice is the $|n,\,0\rangle$ subspace, ${J}_{n}=d{ {\mathcal E} }_{n}^{\mathrm{(0)}}\frac{n}{\sqrt{4{n}^{2}-1}}$, and ${V}_{n}^{ij}=\frac{4{n}^{2}}{4{n}^{2}-1}\frac{V{a}^{3}}{{r}_{ij}^{3}}$, with ${ {\mathcal E} }_{n}^{\mathrm{(0)}}$ the *n*^th^ microwave’s amplitude, *d* the permanent electric dipole moment, *a* the real lattice constant, and $V=\frac{{d}^{2}}{32\pi {\epsilon }_{0}{a}^{3}}$ [see Supplementary material]. When the synthetic lattice is the $|n,n\rangle$ subspace, ${J}_{n}=d{ {\mathcal E} }_{n}^{\mathrm{(0)}}\frac{n}{\sqrt{2n+1}}$ and ${V}_{n}^{ij}=\frac{2n}{2n+1}\frac{V{a}^{3}}{{r}_{ij}^{3}}$, with $V=\frac{-{d}^{2}}{16\pi {\epsilon }_{0}{a}^{3}}$. For both choices of synthetic lattice sites, ${V}_{n}^{ij}\approx V\frac{{a}^{3}}{{r}_{ij}^{3}}$ is nearly independent of *n* for large *n*. Dipole-induced processes that change the total azimuthal angular momentum are off-resonant and average to zero in the rotating frame. Each tunneling amplitude *J*_*n*_ can be tuned by adjusting the amplitude and phase of ${ {\mathcal E} }_{n}^{\mathrm{(0)}}$. Although set to zero in Eq. (1), an on-site potential can be introduced on the *n*^th^ synthetic lattice site by detuning the (*n* − 1)^th^ and *n*^th^ microwaves. We note that since the molecules are stationary, it is irrelevant whether ${\hat{c}}_{nj}$ are fermionic or bosonic operators. Without loss of generality, we assume they are fermionic.

**Figure 2:**
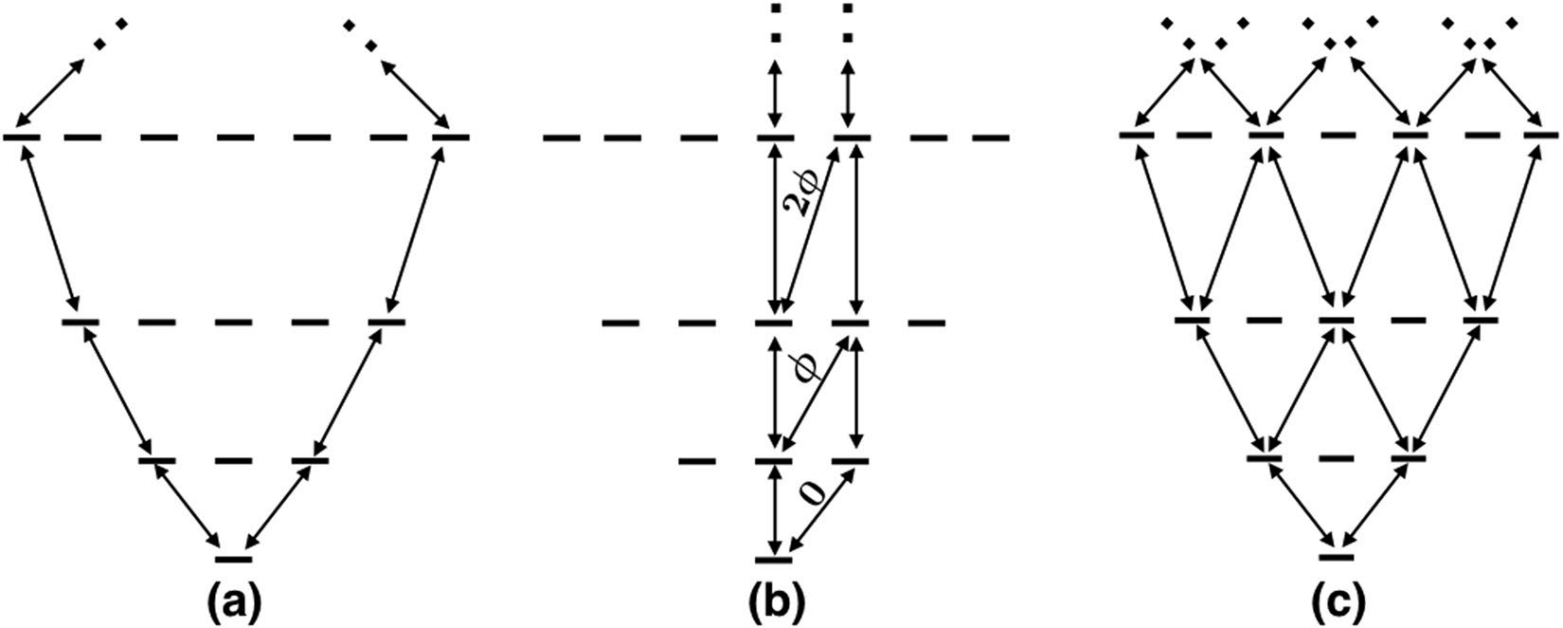
Three examples of engineering a synthetic dimension(s) using the internal rotational states of a molecule, each resulting in a different single-particle Hamiltonian. (**a**) 1D chain with periodic boundaries. (**b**) Two-leg ladder with complex tunnelings. The phase of the tunneling is indicated on the ladder’s rungs. (**c**) Square lattice with open boundaries.

The above setup implements a 1D synthetic lattice with open boundaries. Using a more sophisticated microwave and static field architecture, it is also possible to create arbitrary synthetic lattices, for example a synthetic lattice with periodic boundaries as in Fig. 2(a), a synthetic lattice under a gauge field as in Fig. 2(b), two synthetic dimensions as in Fig. 2(c), or even other topologies. One can also engineer higher lattice connectivities through higher order (e.g. two-photon or three-photon) microwave transitions. We emphasize that this ability to produce a fully controllable sophisticated single particle Hamiltonian is a key advantage of using molecules over atoms.

In this article, we consider the many-body physics in the simplest case of one open-boundary synthetic dimension with uniform and positive *J*_*n*_, and show that dipole interactions lead to rich physics. We assume that the real space lattice is a one-dimensional (1D) chain or a two-dimensional (2D) square lattice. For the square lattice, for the microwave polarizations in our setup, the sign of ${V}_{n}^{ij}$ is isotropic in the real lattice plane. We first determine the two-molecule ground state, then examine the many-body behavior.

## Results

### Bound state of two molecules

We exactly solve Eq. (1) for two molecules. The solution already gives considerable insight into understanding the many-body phase diagram. We analytically solve the problem in the case that *V*_*n*_ = *V* are uniform, and *N*_rot_ is large. We also perform a numerical calculation for finite *N*_rot_ and physical values of *V*_*n*_, and find that the uniform *V*_*n*_ limit captures the essential physics.

**Figure 3:**
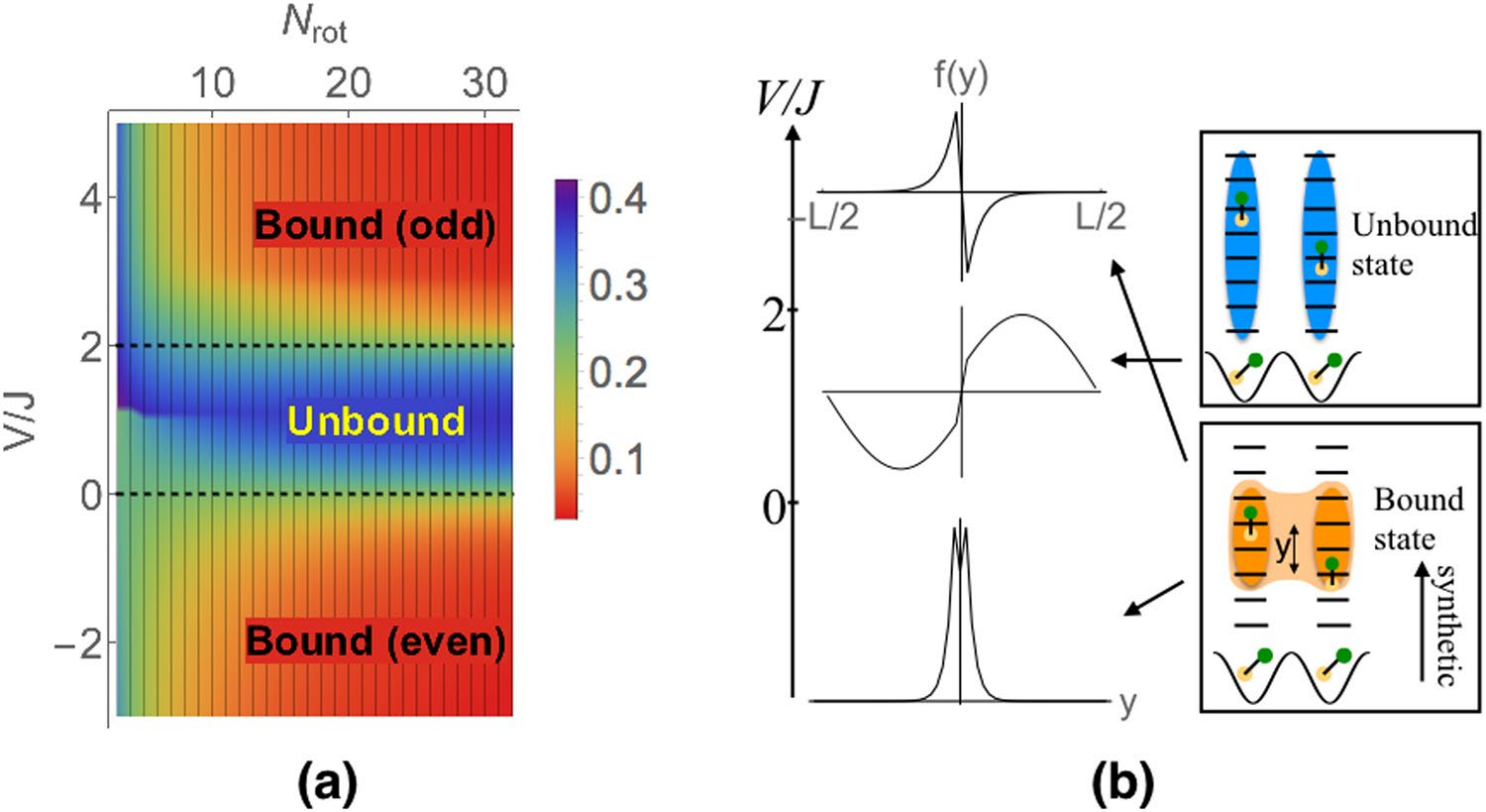
Two-molecule phase diagram. (**a**) Normalized average distance *δ* between the two molecules in the synthetic direction, as a function of interaction strength *V*/*J* and synthetic dimension size *N*_rot_. The ground state undergoes binding transitions at *V* = 0 and *V* = 2*J* (dotted lines) in the thermodynamic limit. (**b**) Representative relative wavefunctions in the three phases.

Writing the most general state for two molecules, $|\psi \rangle ={\sum }_{mn}\,{f}_{mn}{\hat{c}}_{m1}^{\dagger }{\hat{c}}_{n2}^{\dagger }|{\rm{vac}}\rangle$, we find that there are three types of solutions for *f*_*mn*_ in three different regions of parameter space. Figure 3(b) shows the behavior of the ground state in these three regions. For 0 < *V* < 2*J*, the two molecules are in a scattering state, *f*_*mn*_ = *e*^±*i*(*m*−*n*)^ for *m* ≠ *n*, with a scattering phase shift as the molecules cross each other in the synthetic dimension. In this regime, the molecules are delocalized throughout the synthetic direction. If *V*/*J* < 0 or *V*/*J* > 2, the molecules are localized in a bound state, *f*_*mn*_ = *e*^−*λ*(*m*−*n*)^ for *m* > *n*, with a binding length 1/*λ*. The bound state is even under exchange of the two molecules if *V*/*J* < 0, and odd if *V*/*J* > 2. The binding length diverges at the critical points *V*/*J* = 0 and 2, and it monotonically decreases away from these points. As *V*/*J* → ±∞, the two molecules are tightly bound in a state that is two synthetic lattice sites wide:

2 $$|{\psi }_{n}\rangle =\frac{{\hat{c}}_{n\mathrm{,1}}^{\dagger }{\hat{c}}_{n+1,2}^{\dagger }\pm {\hat{c}}_{n+1,1}^{\dagger }{\hat{c}}_{n,2}^{\dagger }}{\sqrt{2}}|{\rm{vac}}\rangle ,$$

where *n* is arbitrary.

The same essential physics exists for finite *N*_rot_ and nonuniform physical couplings ${V}_{n}=V\frac{4{n}^{2}}{4{n}^{2}-1}$. We demonstrate this by numerically diagonalizing Eq. (1). We characterize the ground state by the normalized relative molecular separation in the synthetic direction, $\delta =\frac{1}{{N}_{{\rm{rot}}}}\,{\sum }_{mn}\,|m-n|\langle {\hat{c}}_{m1}^{\dagger }{\hat{c}}_{m1}{\hat{c}}_{n2}^{\dagger }{\hat{c}}_{n2}\rangle$, which we plot in Fig. 3(a). For *N*_rot_ → ∞, *δ* asymptotes to $\frac{{e}^{\lambda }}{2{N}_{{\rm{rot}}}\,\sinh \,\lambda }$ in the bound states, and undergoes a sharp transition at *V* = 0 and *V* = 2*J*. For finite *N*_rot_, the transition is smoother, and occurs at larger |*V*/*J*|.

We emphasize that the ground states of our system differ from conventional magnetically ordered/disordered states of a large-spin system, with the synthetic lattice sites mapped to spin states. In our system, the amplitudes *J*_*n*_ and *V*_*n*_ (for large *n*) are uniform, leading to a translational symmetry in the synthetic direction. This is a highly unnatural Hamiltonian for a large-spin system, and is reflected in the structure of the ground state. The relative width of the ground state in the synthetic direction is finite, unlike ferromagnetic phases that occupy width ${\mathscr{O}}(\sqrt{{N}_{{\rm{rot}}}})$.

### Many-body phase diagram

Next we explore Eq. (1) for 1D and 2D periodic real space arrays of molecules. For a 1D array, we assume a variational ansatz that reproduces the exact solution for two molecules:

3 $$|{\psi }_{{\rm{var}}}\rangle =\prod _{i\in {\rm{even}}}\,\sum _{mn}\,{f}_{mn}{\hat{c}}_{mi}^{\dagger }{\hat{c}}_{n,i+1}^{\dagger }|{\rm{vac}}\rangle \mathrm{.}$$

This ansatz can also be viewed as a “cluster mean field” approximation^54^ on which the system is divided into pairs of sites in real space that are coupled through the mean field.

**Figure 4:**
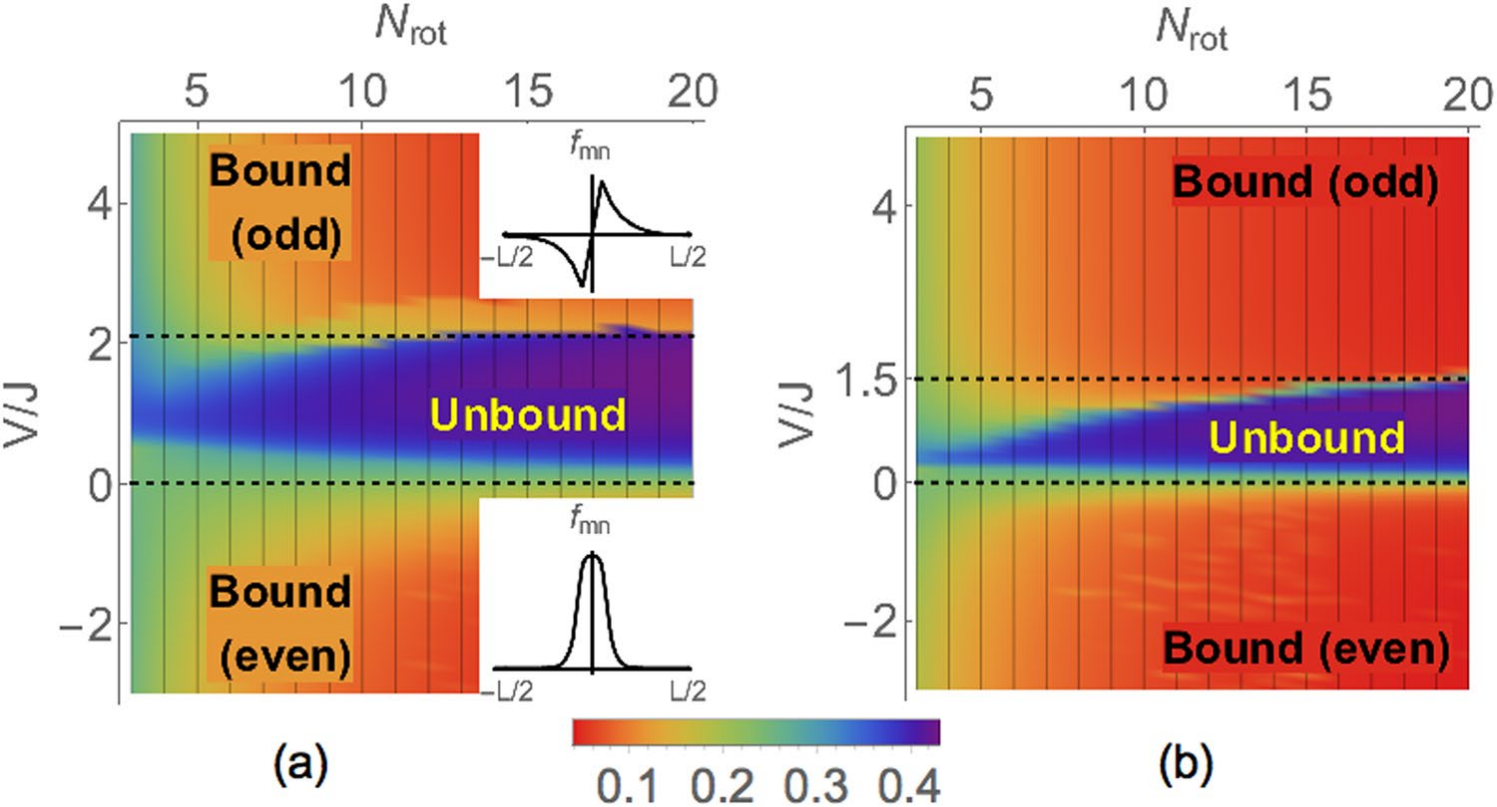
Many-body phase diagram for (**a**) a one-dimensional chain, and (**b**) a square lattice in real space. When $V\lesssim 0$ or $V\gtrsim 2.15J$ [dotted lines in (**a**)] in a 1D chain of molecules, adjacent molecules bind in the synthetic dimension. Bound pairs of molecules align and collapse to a quantum string. The color scale denotes the normalized average synthetic distance *δ* between molecules in a bound pair. For a 2D array, the molecules collapse to a membrane when $V\lesssim 0$ or $V\gtrsim 1.5J$ [dotted lines in (**b**)]. In the intermediate regime, the molecules form a gas. Insets: Representative variational wavefunctions in the quantum string phases.

Figure 4(a) shows the phase diagram found by minimizing the energy with respect to the variational parameters *f*_*mn*_. There are three phases of matter, each corresponding to a type of two-body state found above. In the bound phases, pairs of adjacent molecules bind together in the synthetic direction. Adjacent bound pairs similarly attract each other in this direction. As a result, the system spontaneously collapses to a one-dimensional string. The width of the string in the synthetic dimension varies with *V*/*J*, from two sites wide at *V*/*J* → ±∞, to a diverging value at the transitions. When ${N}_{{\rm{rot}}}\gg 1$, the transitions occur at *V* = 0 and *V* = 2.15*J*. The molecules are unbound when 0 < *V* < 2.15*J*. The transitions are smoothed out and shifted at finite *N*_rot_.

When *V*/*J* = ±∞, the system spontaneously breaks the synthetic translational symmetry and a *U*(1) symmetry of the Hamiltonian. Each molecule along the quantum string spontaneously localizes to only two states $|n\rangle$ and $|n+1\rangle$, forming a string that is two synthetic sites wide, breaking the synthetic translational symmetry. The system can be effectively thought to host hardcore bosons, with $|n\rangle$ corresponding to a vacant site and $|n+1\rangle$ to a singly occupied site. In this description, dipole interactions look like tunneling for the hardcore bosons between real lattice sites, leading to a hardcore Bose-Einstein condensate living on the string as the ground state, with a corresponding broken *U*(1) symmetry.

We also consider a 2D array of molecules in the *x*-*y* plane. We extend the mean field ansatz in Eq. (3) to 2D, and variationally minimize the energy to calculate the phase diagram, which we plot in Fig. 4(b). We again find binding transitions at $V\lesssim 0$ and $V\gtrsim 1.5J$, beyond which the molecules form a quantum membrane. We again find condensate transitions at *V*/*J* = ±∞.

To assess the accuracy of our approximation, we investigate two other variational ansatzes: a single-site mean field, and a mean field theory of fermionic pairs. We find that the phase diagram in all cases is similar to the cluster mean field results. The broad concurrence of these results from very different approximations gives us confidence in the basic physics of our model. In all cases, there are three phases: two phases with molecules forming bound states, and one of unbound molecules. Only the details of the long-range correlations differ among the approximations. We describe our other variational ansatzes in detail in the Supplementary Material.

Although earlier works^55–58^ have obtained broadly similar bound states in models without synthetic dimensions, the situation presented here has experimental advantages and differs in some of the relevant physics. By utilizing a synthetic dimension, string states appear without needing molecules to tunnel in real space, thus avoiding the chemical reactions^18,42–46^ and complex collisional processes^47–52^ that will occur in other proposals and almost surely lead to significant loss or heating. Additionally, the energy scales are more favorable in our proposal. While earlier proposals required the motional degrees of freedom to be cooled below the dipole interaction scale to observe strings, our proposal only requires the rotational state temperature to be below this scale. Experiments routinely achieve near-zero entropy rotational state superpositions, which satisfies our requirement. The phases in the present system also somewhat differ from earlier proposals. First, a condensate lives on them, and, second, we show that one can obtain membranes, which have not appeared in previous work.

## Discussion

### Experimental detection

The phases in our system can be detected by local unitary transformations of rotational states and measurements of ground state populations. Populations in all rotational states can be selectively measured by a direct absorptive image^59^ or a time-resolved image taken with a resonantly enhanced multiphoton ionization^60^. The binding between the molecules can be detected by measuring the distribution of molecules in the synthetic dimension. In the bound phase, adjacent molecules are relatively close to each other in the synthetic dimension. Specifically the relative spread *δ* of molecules in the synthetic dimension is ${\mathscr{O}}\mathrm{(1)}$. In the unbound phase, $\delta \sim {\mathscr{O}}({N}_{{\rm{rot}}})$ is large. The even/odd parity of the bound state and the *U*(1) symmetry breaking can be characterized by the single-particle coherences between rotational states, which can be measured by performing *π*/2 unitary transformations between the desired rotational states before measuring populations.

### Variations of the experimental setup

The full control over the single particle Hamiltonian in our system results in the potential to explore a wide variety of other physics. For example, staggering the amplitudes *J*_*n*_ would lead to a topological band structure^61–64^. Randomizing the microwave detunings – which we set to zero in Eq. (1) – or the tunneling amplitudes would allow us to explore physics related to Anderson and many-body localization^27,65^. Periodic modulation of the microwave amplitudes or frequencies will allow us to realize effective interactions between three or more particles in the Floquet picture, which will result in novel physics^66^. Engineering a two-dimensional synthetic lattice with complex tunneling amplitudes would implement synthetic gauge fields, manifesting for instance the Hofstadter butterfly^67,68^. Reintroducing tunneling between real lattice sites also allows access to a rich variety of physics, extending the *N*_rot_ = 2 case studied in refs^9,10,12^. In the presence of real-space tunneling, multiple molecules can occupy the same lattice site in the lowest band, and dipole interaction produces a synthetic nearest-neighbor interaction between molecules that lie on the same real lattice site. These nearest-neighbor interactions lead to charge density wave or *p*-wave superfluid order in the synthetic direction^69–71^.

## Summary

We have shown that the rotational states of polar molecules can act as a highly controllable synthetic dimension. The tunnelings and band structure in this dimension can be precisely tuned by arbitrary microwave waveforms, and probed by established spectroscopic tools. This opens avenues to explore physics related to Anderson and many-body localization, synthetic gauge fields, topological band structures, and topological superfluids. The unusual form of dipole interactions in the synthetic dimension opens up new types of phenomena to ultracold matter.

We examined the many-body physics arising from interactions between polar molecules, in the simplest experimental scenario where microwaves drive tunneling of molecules in a synthetic dimension with a uniform amplitude, and where real space tunneling is suppressed. We found intriguing forms of correlated quantum matter, including fluctuating quantum strings and membranes, on whose surfaces live strongly interacting condensates. We emphasize that this physics emerges naturally with no intricate engineering or fine tuning, and the energy scales are large – set directly by the dipole interaction. Moreover, because our setup avoids double occupancies in the real space lattice, the experimental lifetime is much longer than the ~ms timescales associated with the dipole interaction. It will be fascinating for future research to explore these membranes, the interplay between the quantum fluctuations of the membrane and the condensate that lives on it, and their stability in the presence of perturbations such as disorder.

## Electronic supplementary material

Supplementary material
