# Supplementary material for: Synthetic dimensions in ultracold polar molecules
Source: Sci Rep. 2018 Feb 21;8:3422. doi: 10.1038/s41598-018-21699-x (PMC5821820; doi:10.1038/s41598-018-21699-x)
Supplement: Supplementary file 1 — Supplementary material [file 41598_2018_21699_MOESM1_ESM.pdf]

Supplementary Materials for *Synthetic dimensions in ultracold polar molecules*

Bhuvanesh Sundar,[1,2,*] Bryce Gadway,[3,†] and Kaden R. A. Hazzard[1,2,‡]

[1]*Department of Physics and Astronomy, Rice University, Houston TX 77251, USA*
[2]*Rice Center for Quantum Materials, Rice University, Houston TX 77251, USA*
[3]*Department of Physics, University of Illinois at Urbana Champaign, Urbana IL 61801, USA*

EFFECTIVE HAMILTONIAN

In the lab frame, our system is described by the Hamiltonian

$$\hat{H} = \sum_j B\hat{N}_j^2 + \sum_j \sum_{n=1}^{N_{\rm rot}} \hat{\vec{d}}_j \cdot \vec{\mathcal{E}}_n(t) + \sum_{ij} \frac{\hat{\vec{d}}_i \cdot \hat{\vec{d}}_j - 3\left(\hat{\vec{d}}_i \cdot \hat{r}_{ij}\right)\left(\hat{\vec{d}}_j \cdot \hat{r}_{ij}\right)}{4\pi\epsilon_0 r_{ij}^3}, \tag{S1}$$

where $\hbar\hat{N}_j$ is the rotational angular momentum operator for the $j^{\rm th}$ molecule, and $B \sim \hbar \times \mathcal{O}\,({\rm GHz})$ is the rotational constant. The eigenstates $|n, m\rangle$ of the first term in Eq. (S1) are described by two quantum numbers: the angular momentum quantum number n and its projection m on the z axis. Equation (S1) assumes that the molecules are rigid rotors, i.e all molecules are in the lowest vibrational state, and coupling to excited vibrational states is small. This assumption is valid up to n that approximately lies in a range between 40 and 60 in a typical diatomic molecule. (For example, for RbCs whose rotational constant is $\sim h \times 500$ MHz and vibrational excitation energy is $\sim h \times 1.5$ THz, the rotational excitation above the ground state is smaller than the vibrational excitation for $n \lesssim 55$. For NaRb, whose rotational constant and vibrational excitation energy are $\sim h \times 2$ GHz and $\sim h \times 3.2$ THz, the rotational excitation above the ground state is smaller than the vibrational excitation for $n \lesssim 40$). For larger n, the rotational states get dressed by rovibrational coupling.

The matrix elements for the dipole moment operator between rotational states in a rigid rotor are [1, 2]

$$\begin{aligned}\langle n, m|\, \hat{d}^p \,|n', m'\rangle =& (-1)^p d\sqrt{\frac{4\pi}{3}} \int\int Y_{n'm'}(\Theta, \Phi) Y^*_{nm}(\Theta, \Phi) Y_{1p}(\Theta, \Phi) \sin\Theta d\Theta d\Phi \\ =& (-1)^p d\sqrt{\frac{2n+1}{2n'+1}} \langle n, m; 1, p|n', m'\rangle \langle n, 0; 1, 0|n', 0\rangle\,,\end{aligned} \tag{S2}$$

where $\hat{d}^p$ are the spherical components of the dipole operator, d is the molecule's permanent electric dipole moment, and p takes the values $0, \pm 1$. Using the properties of Clebsch-Gordan coefficients, Eq. (S2) simplifies to

$$\begin{aligned}\langle n, m|\, \hat{d}^0 \,|n-1, m'\rangle &= d\delta_{mm'}\sqrt{\frac{n^2-m^2}{4n^2-1}} \\ \langle n, m|\, \hat{d}^\pm \,|n-1, m'\rangle &= \pm d\delta_{m,m'\pm 1}\sqrt{\frac{(n\pm m)(n\pm m-1)}{2(4n^2-1)}} \\ \langle n, m|\, \hat{d}^p \,|n', m'\rangle &= 0 \text{ if } |n-n'| \neq 1.\end{aligned} \tag{S3}$$

For larger n where the rigid rotor assumption breaks down, the polarization of the molecules typically reduces due to a larger internuclear separation, so the matrix elements are smaller than Eq. (S3). In this limit, matrix elements can be obtained from techniques in earlier works [3] which calculate dipole moments as a function of the internuclear separation.

We find from Eqs. (S1) and (S3) that when the molecules are illuminated by a resonant $\hat{z}$-polarized microwave of amplitude $\mathcal{E}_n^{(0)}$, the synthetic tunneling strength from $|n-1, 0\rangle$ to $|n, 0\rangle$ is $J_n = d\mathcal{E}_n^{(0)} \frac{n}{\sqrt{4n^2-1}}$. When the molecules are illuminated by a σ^+-polarized microwave, the tunneling strength from $|n-1, n-1\rangle$ to $|n, n\rangle$ is $J_n = d\mathcal{E}_n^{(0)}\sqrt{\frac{n}{2n+1}}$. We choose the microwave fields such that $J_n = J$ is uniform in n: $\mathcal{E}_n^{(0)} = \frac{J\sqrt{4n^2-1}}{nd}$ for the $m = 0$ states, and $\mathcal{E}_n^{(0)} = \frac{J\sqrt{2n+1}}{\sqrt{n}d}$ for $m = n$. Even when the dipole matrix elements are smaller than Eq. (S3) due to rovibrational

mixing, the synthetic tunnelings can still be made uniform by compensating with stronger microwaves. To tune this, experiments can perform Rabi spectroscopy on the relevant two level system, and adjust the microwave amplitude until the desired tunneling rate is achieved.

Dipole interactions induce an angular momentum exchange with an amplitude V_n^{ij}, between two molecules in adjacent rotational manifolds. When the two molecules are in $m=0$ states, $V_n^{ij} = \langle n-1,0|_i \langle n,0|_j \hat{H}_{\rm dd} |n,0\rangle_i |n-1,0\rangle_j = \frac{1-3(\hat{r}_{ij}\cdot\hat{z})^2}{8\pi\epsilon_0 r_{ij}^3}\frac{d^2n^2}{4n^2-1}$, where $\hat{H}_{\rm dd}$ is the third term in Eq. (S1). For the $m=n$ states, $V_n^{ij} = \frac{3(\hat{r}_{ij}\cdot\hat{z})^2-1}{8\pi\epsilon_0 r_{ij}^3}\frac{d^2n}{2n+1}$. These expressions are modified at large n, when rovibrational mixing is relevant [3].

Interactions between the rotational and nuclear spin angular momenta introduce some mixing between the molecule's rotational and hyperfine spin quantum numbers. However, because we add a small electric field $\sim \mathcal{O}(10)$ V/cm, the energies of the different m states within a rotational manifold split by $\sim$ 10-100 MHz. This splitting is typically larger than the interaction between nuclear and rotational angular momentum. As a result, the rotational angular momentum decouples from the nuclear spins, so m is a good quantum number [4]. The expressions for the matrix elements for the dipole moment operator [Eq. (S3)] are valid in this limit. Additionally, the splitting of the m states within a rotational manifold is also larger than V_n^{ij}. Therefore, all dipole interaction-induced transitions to rotational states outside the Hilbert space in Eq. (1) are off-resonant and suppressed. The electric field does mix states between different rotational manifolds n, but the resulting additions to the Hamiltonian in Eq. (1) are insignificant for these electric field values.

MICROWAVE IMPLEMENTATION

Here, we briefly discuss the implementation of microwaves for the realization of the Hamiltonian in Eq. (1), and associated challenges. As a concrete example, we consider RbCs, whose rotational constant is $B \approx h \times 500$ MHz. Cold and dense samples of RbCs have been produced by both the Innsbruck [5, 6] and Durham [7] groups. The microwave frequency required to excite a molecule from the $|n-1\rangle$ to the $|n\rangle$ rotational manifold is $\omega_n = 2nB/\hbar \approx 2\pi n$ GHz. We consider the scenario where only the $m=0$ rotational sublevels are populated, such that the number of synthetic lattice sites will be equal to the number of coupled rotational manifolds. In this case, an implementation of a 1D synthetic lattice with $N_{\rm rot}$ synthetic lattice sites and open boundary conditions requires $N_{\rm rot}-1$ microwaves, spanning (sparsely) the range from 1 GHz to $(N_{\rm rot}-1)$ GHz.

The generation, amplification, and projection of microwaves to coherently couple a large number of synthetic sites can be straightforwardly accomplished using standard and commercially available microwave equipment. The required microwave waveform itself, $\mathcal{E}(t) = \sum_{n=1}^{N_{\rm rot}-1}\mathcal{E}_n^{(0)}\cos(\omega_n t)$, can be generated with lab-developed or commercially available arbitrary waveform generators presently capable of reaching up to at least 20 GHz [8]. Additionally, since only a finite number of discrete frequencies are actually required from the large frequency span, versatile frequency sources generated by direct digital synthesis may be used to stroboscopically sample the various ω_n. Standard broadband amplifiers and waveguide horns can then be used to project these signals onto the molecular samples.

At least for some molecules like RbCs, dozens of coupled synthetic sites should be straightforward to realize with present microwave technology. The number of coupled synthetic sites is fewer for molecules which have a larger rotational constant; for example, the synthetic lattice will contain roughly ten sites for NaRb or KRb. In addition to being limited by the frequency range of waveform generators, the number of coupled synthetic sites is also limited by the lifetime of excited rotational states. The decay rate of a molecule with typical parameters ($B = h\times 500$ MHz and $d = 1$D), from $n=20$ due to stimulated emission by a microwave field at room temperature, is $\sim 1/$ sec. The decay rate is larger at larger n. Therefore for decay to be insignificant on an experimental timescale$\sim$ s, the number of coupled synthetic sites in this molecule is $n \lesssim 20$. Even so, the synthetic dimension obtained here is significantly larger than can be obtained with alkali atoms.

Furthermore, a synthetic dimension of even larger sizes, up to hundreds of synthetic lattice sites, can be obtained using essentially the same equipment and approach, if we remove the restriction of using only the $m=0$ or $m=n$ rotational sublevels; unique spectral selectivity of state-to-state transitions is generally still provided by rotational-hyperfine energy shifts at the scale of $\sim$ 100 kHz.

The biggest challenge in the implementation of our scheme arises from the fact that different rotational states may experience different real space lattice potentials due to having different ac polarizabilities. This has two consequences: molecules in different rotational states have a) different zero point energies, which shift the microwaves off resonance, and b) different real space Wannier functions, which affect dipole matrix elements. The offsets in the zero point energies can be cancelled by tuning the microwave frequencies appropriately, and/or choosing "magic angles" for the polarization of the lattice lasers [9, 10]. It may be more practical to tune the frequencies to resonance, because

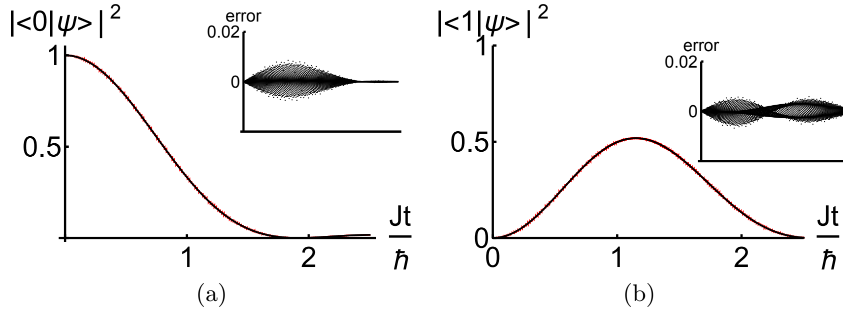

FIG. S1. The occupation probability of the (a) $|n=0\rangle$ and (b) $|n=1\rangle$ synthetic lattice sites versus time, when the molecule is initialized in $|n=0\rangle$, and evolves under the tight-binding Hamiltonian that is the first term in Eq. (1) of the main text (black) or under the single-particle terms in Eq. (S1) (red) with 100 microwaves. The black and red curves are indistinguishable by eye at the scale shown, demonstrating that the tight binding model accurately describes the molecule's evolution when it is driven by 100 microwaves. The microwave amplitudes were chosen such that $J = B/200$. This is a pessimistically large tunneling; the tight-binding model becomes more accurate as J/B is decreased. We expect that the typical synthetic tunneling will be $J/B \sim 10^{-6}$, as explained in the text. Inset: Difference between black and red curves.

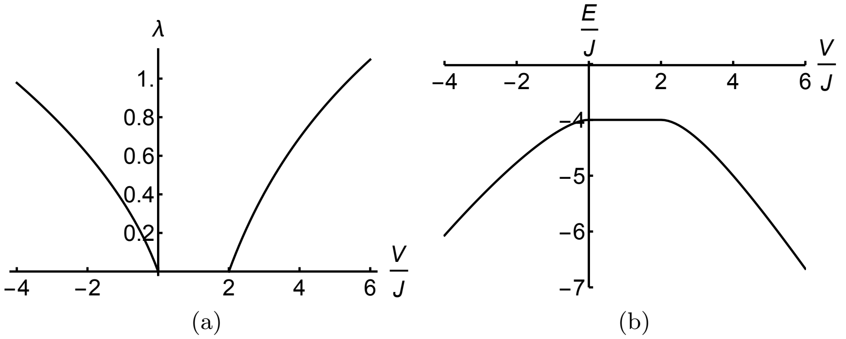

FIG. S2. (a) The inverse binding length as a function of V/J, where V refers to the amplitude for exchanging angular momentum at large n. The molecules undergo a transition from an unbound state to a bound state at $V/J = 0$ and $V/J = 2$. (b) The ground state energy versus V/J.

the polarizabilities are very sensitive to small deviations in the polarization angles. The effect on the dipole matrix elements can be compensated by adjusting the respective microwave amplitude until the desired matrix elements for a uniform synthetic tunneling are achieved. The only remaining challenge is then due to inhomogeneities in the real space optical lattice, and a harmonic trap in real space, which will lead to a spatially varying zero point energy. This challenge can be mitigated by using a flat trap, or by observing only a small region of the molecular cloud in a harmonic trap where the optical lattice is relatively uniform in real space. In such a small region, all microwaves can be tuned to resonance with the desired rotational transitions.

One might worry that the illumination of the cloud with a large number of microwaves presents an additional challenge: that the numerous off-resonant driving frequencies lead the molecules to decohere, and that the tight binding model Eq. (1) is no longer valid. However, there is no such decoherence, due to a large separation of energy scales between the microwave frequencies and microwave couplings. For example, a typical microwave frequency used is $\omega_n \sim \mathcal{O}(\mathrm{GHz})$, and a typical synthetic tunneling, for moderate microwave amplitude, is $J \sim \hbar \times \mathcal{O}(\mathrm{kHz})$. For these values, the dynamics of the molecules are modeled accurately by the tight-binding model in Eq. (1). We illustrate this in Fig. S1, by comparing the dynamics of a molecule governed by [Eq. (1)], and one governed by the single particle terms in Eq. (S1) which include the 100 microwaves required to create a synthetic dimension with 101 sites. We find that even for very large synthetic tunnelings $J \sim \frac{\hbar\omega_n}{200}$, the dynamics in the two cases agree to better than 1% accuracy.

BOUND STATE FOR TWO MOLECULES

The Schrödinger equation for the relative wavefunction of two molecules, $f(y = m - n) = f_{mn}$, is

$$\begin{aligned} Ef(y) &= -J\left[f(y+1)+f(y-1)\right] && \text{if } |y| \neq 1 \\ Ef(y) &= -J\left[f(y+1)+f(y-1)\right]+Vf(-y) && \text{if } |y| = 1. \end{aligned} \tag{S4}$$

We substitute the ansatz that $f(y) = e^{-\lambda y}$ for $y > 0$. The first line in Eq. (S4) tells us that the energy of this bound state is $E = -4J \cosh\lambda$. When $V \leq 0$, we find that the ground state satisfies $f(y) = f(-y)$. Using the second line in Eq. (S4), we find that λ solves the implicit transcendental equation:

$$e^{\lambda} = \frac{2J}{V + 4J\cosh\lambda - 2J\,\mathrm{sech}\,\lambda}. \tag{S5}$$

The solution for λ in Eq. (S5) is positive for all $V \leq 0$. When $V > 0$, we find that in the ground state, $f(y) = -f(-y)$. In this case, λ solves the transcendental equation:

$$e^{\lambda} = \frac{-2J}{V - 4J\cosh\lambda}. \tag{S6}$$

The solution for λ in Eq. (S6) is positive only when $V \geq 2J$. The positive solution for λ, and the ground state energy, are plotted in Fig. S2.

OTHER VARIATIONAL APPROXIMATIONS

In the main text we presented the results for the many-body phase diagram based on a cluster mean field theory. Here we consider two alternative approximations that corroborate the findings there.

Single-site mean field approximation

Our single-site mean field ansatz for a 1D chain of molecules is

$$|\psi'_{\rm var}\rangle = \left(\prod_{i\in\text{even}}\sum_m g_m \hat{c}^\dagger_{mi}\right)\left(\prod_{j\in\text{odd}} h_n \hat{c}^\dagger_{nj}\right)|\text{vac}\rangle\,. \tag{S7}$$

The variational energy per molecule in this approximation is

$$\begin{aligned} \frac{\left\langle \hat{H}\right\rangle'_{\rm var}}{N_{\rm mol}} = &-\frac{J}{2}\sum_m\left(g^*_m g_{m+1} + h^*_m h_{m+1} + \text{c.c.}\right) + \frac{7\zeta(3)}{8}\sum_n V^{01}_n\left(g^*_n g_{n+1} h^*_{n+1} h_n + \text{c.c.}\right) \\ &+ \frac{\zeta(3)}{8}\sum_n V^{01}_n\left(|g_n g_{n+1}|^2 + |h_n h_{n+1}|^2\right) \end{aligned} \tag{S8}$$

Similar to the cluster mean field approximation in the main text, we found that this ansatz also predicts two binding transitions, which occur at $V \simeq 0$ and $V \simeq 2.15J$ in the thermodynamic limit. The binding transitions again correspond to a spontaneous dimensional reduction to a fluctuating quantum string. The string hosts an emergent hardcore condensate at $|V/J| = \infty$.

The variational ansatz for a square lattice of molecules is obtained by a straightforward extension of the 1D ansatz in Eq. (S7). That is, bipartition the square lattice into two intercalated checkerboards in the conventional way, run the index i over one checkerboard, and j over the other checkerboard. Here again, we found that two binding transitions occur, similar to the cluster approximation. When $V \simeq 0$ or $V \simeq 1.5J$, the system undergoes a spontaneous dimensional reduction to a fluctuating quantum membrane. The membrane hosts an emergent hardcore condensate at $|V/J| = \infty$.

Fermionic-pair mean field approximation for a 1D chain

In this approximation, we generalize our system to a grand canonical ensemble of slave fermions, by introducing a chemical potential. The modified Hamiltonian that describes our system is

$$\hat{H}_{\rm eff} = \sum_j \sum_{n=1}^{N_{\rm rot}} \left(-J_n \left(\hat{c}^\dagger_{n-1,j}\hat{c}_{nj} + \text{h.c.}\right) - \mu \hat{c}^\dagger_{nj}\hat{c}_{nj}\right) + \sum_{ij}\sum_{n=1}^{N_{\rm rot}} V_n^{ij}\left(\hat{c}^\dagger_{ni}\hat{c}_{n-1,i}\hat{c}^\dagger_{n-1,j}\hat{c}_{nj} + \text{h.c.}\right). \tag{S9}$$

We relax the constraint that exactly one molecule occupies each real lattice site, and fix the chemical potential μ such that this constraint is held on average: $\sum_n \left\langle \hat{c}^\dagger_{nj}\hat{c}_{nj}\right\rangle = 1\ \forall j$. Motivated by our exact solution for two molecules, we assume mean fields for bound molecules in the synthetic dimension:

$$\Delta_n^{ij} = V_n^{ij}\left\langle \hat{c}_{nj}\hat{c}_{n-1,i}\right\rangle. \tag{S10}$$

To gain a simple understanding, we assume that $V_n^{ij} = V^{ij}$ and $J_n = J > 0$ are uniform in n. Further, we consider only nearest-neighbor interactions, $V^{ij} = V\delta_{|i-j|,1}$, and periodic boundary conditions in both real and synthetic dimensions. Later we perform a numerical calculation, where we include nonuniform physical values V_n in the low-lying rotational states, and open boundary conditions.

In the fermion-pair approximation, the mean-field Hamiltonian for a 1D chain of molecules is

$$\hat{H}_{\rm MF} = \sum_{nj}\left(-J\left(\hat{c}^\dagger_{nj}\hat{c}_{n-1,j} + \text{h.c.}\right) - \mu\hat{c}^\dagger_{nj}\hat{c}_{nj} + \left(\Delta \hat{c}^\dagger_{nj}\hat{c}^\dagger_{n-1,j+1} + \Delta'\hat{c}^\dagger_{n,j+1}\hat{c}^\dagger_{n-1,j} + \text{h.c.}\right)\right). \tag{S11}$$

Here, $\Delta = \Delta_n^{j,j+1}$ and $\Delta' = \Delta_n^{j+1,j}$. We self-consistently diagonalize $\hat{H}_{\rm MF}$ to find Δ, Δ' and μ. We find different solutions when $V < 0$ and $V > 0$. In both regimes, the parameters Δ, Δ' and μ solve the implicit equations:

$$\frac{1}{|V|} = \frac{2}{N_{\rm rot}N_{\rm mol}}\sum_{\vec{k}} \frac{\left|g^2(\vec{k})\right|}{\sqrt{(\mu + 2J\cos k_Z)^2 + 16\Delta^2\left|g^2(\vec{k})\right|}}, \tag{S12}$$

$$1 = \frac{1}{2N_{\rm mol}}\sum_{\vec{k}}\left(1 + \frac{\mu + 2J\cos k_Z}{\sqrt{(\mu + 2J\cos k_Z)^2 + 16\Delta^2\left|g^2(\vec{k})\right|}}\right). \tag{S13}$$

When $V < 0$, $\Delta = \Delta'$ and $g(\vec{k}) = \cos k_Z \sin k_X$ is a self-consistent solution. When $V > 0$, $\Delta = -\Delta'$ and $g(\vec{k}) = \sin k_Z \cos k_X$ is a self-consistent solution. The free energy per molecule is

$$\frac{\left\langle \hat{H}\right\rangle_{\rm MF}}{N_{\rm mol}} = -\frac{1}{2N_{\rm mol}}\sum_{\vec{k}}\left(\mu + 2J\cos k_Z - \frac{4|\Delta|^2}{|V|} + \sqrt{(\mu + 2J\cos k_Z)^2 + 16\Delta^2 g^2(\vec{k})}\right). \tag{S14}$$

Here, X and Z refer to the real and synthetic directions (not the same as x and z), and k_X and k_Z are momenta conjugate to X and Z. The mean field ground states are achiral p-wave superfluids of slave fermions. The order parameter is even (odd) along the synthetic direction, and odd (even) along the real direction, when $V < 0$ ($V > 0$). This is related to the parity of the two-molecule ground state when $V < 0$ and $V > 0$. In the thermodynamic limit of $N_{\rm rot}$ and $N_{\rm mol}$, the system is in the superfluid phase for $V < 0$ and $V > 2J$. For $N_{\rm rot} \gg 1$ and $V/J \to \pm\infty$, the mean field superfluids are identical to the two-molecule ground state in Eq. (2) when they are projected onto the physical sector of unit molecular filling. The system undergoes a phase transition to a normal Fermi liquid phase at $V = 0$ and $V = 2J$.

Fermionic-pair mean field approximation in 2D: chiral superfluids

In a 2D array of molecules, the fermionic-pair mean field approximation results in chiral as well as achiral superfluid phases. The mean-field Hamiltonian for a 2D array, assuming only nearest-neighbor dipole interactions

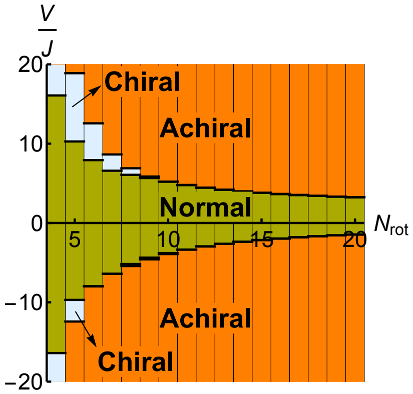

FIG. S3. Phase diagram of a 2D array of molecules under a fermionic-pair mean field approximation. Chiral $p_x + ip_y$ superfluids emerge in a narrow region of V/J for small N_{rot}.

$V_n^{ij} = V\delta_{|i-j|=1}$, is

$$\hat{H}_{\mathrm{MF}} = \sum_{nj}\Big(-J\left(\hat{c}_{nj}^\dagger \hat{c}_{n-1,j} + \mathrm{h.c.}\right) - \mu \hat{c}_{nj}^\dagger \hat{c}_{nj} + \left(\Delta_X \hat{c}_{nj}^\dagger \hat{c}_{n-1,j+\hat{X}}^\dagger + \Delta'_X \hat{c}_{n,j+\hat{X}}^\dagger \hat{c}_{n-1,j}^\dagger + \Delta_Y \hat{c}_{nj}^\dagger \hat{c}_{n-1,j+\hat{Y}}^\dagger + \Delta'_Y \hat{c}_{n,j+\hat{Y}}^\dagger \hat{c}_{n-1,j}^\dagger + \mathrm{h.c.}\right)\Big), \tag{S15}$$

where $\Delta_A = \Delta_n^{j,j+\hat{A}}$ and $\Delta'_A = \Delta_n^{j+\hat{A},j}$ ($\hat{A} = \hat{X}$ or $\hat{Y}$). When the dipole interactions V_n are equal and negative in both real directions, there are two allowed self-consistent solutions:

$$\begin{aligned} &\Delta_X = \Delta'_X = \Delta_Y = \Delta'_Y, \quad && g(\vec{k}) = \cos k_Z \left(\sin k_X + \sin k_Y\right), \text{ or} \\ &\Delta_X = \Delta'_X = i\Delta_Y = i\Delta'_Y, \quad && g(\vec{k}) = \cos k_Z \left(\sin k_X + i \sin k_Y\right). \end{aligned} \tag{S16}$$

The first solution in Eq. (S16) describes an achiral superfluid, while the second solution is chiral. When the dipole interactions V_n are equal and positive in both real directions, there are two allowed self-consistent solutions:

$$\begin{aligned} &\Delta_X = -\Delta'_X = \Delta_Y = -\Delta'_Y, \quad && g(\vec{k}) = \sin k_Z \left(\cos k_X + \cos k_Y\right), \text{ or} \\ &\Delta_X = -\Delta'_X = i\Delta_Y = -i\Delta'_Y, \quad && g(\vec{k}) = \sin k_Z \left(\cos k_X + i \cos k_Y\right). \end{aligned} \tag{S17}$$

The first solution again describes an achiral superfluid, while the second solution is chiral.

We calculate the energy of the chiral and achiral superfluids from Eq. (S14), and plot the superfluid phase with the least energy in Fig. S3. We find that the chiral phase is the most stable superfluid in a narrow region of V/J for small N_{rot}. When projected to the physical subspace, the chiral superfluids correspond to non-Abelian Ising anyonic phases [11].

Comparison of energy in various approximations

In a 1D chain, the total variational energy per molecule in the cluster mean field approximation [Eq. (3)] is

$$\frac{\left\langle \hat{H} \right\rangle_{\mathrm{var}}}{N_{\mathrm{mol}}} = \Bigg(-\frac{J}{2}\sum_{mn} f_{mn}^* \left(f_{m,n+1} + f_{m+1,n}\right) + \frac{1}{2}\sum_n V_n^{01} f_{n,n+1}^* f_{n+1,n} + \frac{7\zeta(3)-4}{8}\sum_{mm'n} V_n^{01} f_{mn}^* f_{n+1,m'}^* f_{m,n+1} f_{n,m'} + \frac{\zeta(3)}{16}\sum_{mm'n} V_n^{01}\left(f_{mn}^* f_{m',n+1}^* f_{m,n+1} f_{m',n} + f_{nm}^* f_{n+1,m'}^* f_{n+1,m} f_{n,m'}\right)\Bigg) + \mathrm{c.c.} \tag{S18}$$

The 1D variational energy in the single-site and fermionic-pair mean field approximations were given in Eqs. (S8) and (S14). For simplicity, in this section, we modify the expressions to include only nearest-neighbor dipole interactions, and plot the variational minimum in the three approximations in Fig. S4.The physics is unchanged when the

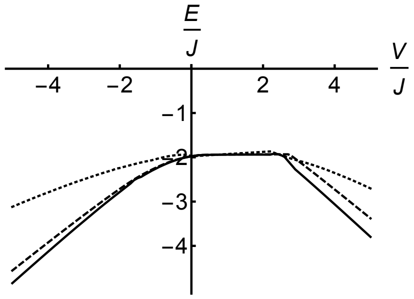

FIG. S4. The ground state energy for a 1D chain of molecules in three different approximations: a cluster mean field approximation (solid), single-site mean field (dashed), and fermion-pair mean field (dotted). The calculation was performed with only nearest-neighbor dipolar interactions and the physical values of V_n. The size of the synthetic dimension was chosen to be $N_{\rm rot} = 20$. The cluster mean field state always has the lowest energy. The energies are similar when the full long-ranged dipolar interaction is included.

full dipole interaction is included. We find that the cluster mean field state always has the lowest energy. The single-site mean field state has nearly the same energy as the cluster mean field state for $V < 2J$, and the fermionic-pair state displays similar behavior as the single-site and cluster mean field states. These observations suggest that all three approximations capture the essential features of the ground state, and the cluster mean field state is the closest approximation to the true ground state.

We also calculated the variational energies of the ground state of a square lattice of molecules in the three approximations. We again found that the cluster mean field state has the lowest energy, and the other approximations result in similar energies.

* bs55@rice.edu
† bgadway@illinois.edu
‡ kaden@rice.edu

[1] J. M. Brown and A. Carrington, *Rotational spectroscopy of diatomic molecules* (Cambridge University Press, 2003).
[2] A. V. Gorshkov, S. R. Manmana, G. Chen, E. Demler, M. D. Lukin, and A. M. Rey, Phys. Rev. A **84**, 033619 (2011).
[3] S. Kotochigova, P. S. Julienne, and E. Tiesinga, Phys. Rev. A **68**, 022501 (2003).
[4] S. A. Will, J. W. Park, Z. Z. Yan, H. Loh, and M. W. Zwierlein, Phys. Rev. Lett. **116**, 225306 (2016).
[5] T. Takekoshi, L. Reichsöllner, A. Schindewolf, J. M. Hutson, C. R. Le Sueur, O. Dulieu, F. Ferlaino, R. Grimm, and H.-C. Nägerl, Phys. Rev. Lett. **113**, 205301 (2014).
[6] L. Reichsöllner, A. Schindewolf, T. Takekoshi, R. Grimm, and H.-C. Nägerl, Phys. Rev. Lett. **118**, 073201 (2017).
[7] P. K. Molony, P. D. Gregory, Z. Ji, B. Lu, M. P. Köppinger, C. R. Le Sueur, C. L. Blackley, J. M. Hutson, and S. L. Cornish, Phys. Rev. Lett. **113**, 255301 (2014).
[8] J. Raftery, A. Vrajitoarea, G. Zhang, Z. Leng, S. J. Srinivasan, and A. A. Houck, arXiv preprint arXiv:1703.00942 (2017).
[9] S. Kotochigova and D. DeMille, Phys. Rev. A **82**, 063421 (2010).
[10] B. Neyenhuis, B. Yan, S. A. Moses, J. P. Covey, A. Chotia, A. Petrov, S. Kotochigova, J. Ye, and D. S. Jin, Phys. Rev. Lett. **109**, 230403 (2012).
[11] F. J. Burnell and C. Nayak, Phys. Rev. B **84**, 125125 (2011).
